# The Golden Ticket? Widening Access in UK Medicine and the Making of an Emotional Proletariat

**DOI:** 10.1111/1467-9566.13860

**Published:** 2024-11-21

**Authors:** Louise Ashley

**Affiliations:** ^1^ School of Business and Management Queen Mary University of London London UK

**Keywords:** Bourdieu, emotional capital, social stratification, socio‐economic diversity, widening participation

## Abstract

‘Widening Access’ in UK medicine seeks to improve access on the basis of socioeconomic background (SEB). However, evidence has emerged of ‘socially stratified’ careers, as doctors from less advantaged backgrounds are more likely to train in less competitive specialties, such as psychiatry or primary care. These patterns have been welcomed to date as this improves access to care, yet less positive consequences have been overlooked. Based on in‐depth interviews (*n* = 54) with medical students, qualified doctors and medical educators from less advantaged backgrounds (*n* = 38), this article asks how values influence medical careers, for what this can tell us about the causes of social stratification and how this informs status hierarchies within the profession. Using the work of Bourdieu, we find that while participants value empathy and compassionate care they believe both are less valuable when securing more competitive careers, and may signal less skill. This helps explain why doctors from less advantaged careers may prefer more community orientated roles, which are often less competitive, and why these specialties may also attract less status and respect. A related risk is that doctors from less advantaged backgrounds are over‐represented in areas imposing the highest emotional demands to become the profession's ‘emotional proletariat’.

## 1 Introduction

While the medical profession has diversified according to gender and ethnicity, currently just over 6% of doctors are from less advantaged socioeconomic backgrounds (SEB), as defined by parental occupation and income (Friedman, Laurison, and Macmillan 2017). The ‘Widening Access’ (WA) agenda is intended to respond to this challenge, leading, for example, to a relative increase of 28% between 2007 and 2016 amongst medical students whose parents do not have higher education qualifications (Medical Schools Council 2018). However, evidence has emerged of socially stratified careers: in other words, doctors from less advantaged SEBs are more likely to train in specialties which have historically been less competitive on entry. For example, one study found that doctors who attended a fee‐paying school (a proxy for advantage in the UK) are 1.7 and 1.4 times more likely to be training for speciality positions in medicine or surgery respectively, compared to general practice (Santana and Chalkley 2017). After controlling for multiple factors, Kumwenda et al. (2019) found that students from families where no parent was educated to degree level had lower odds of choosing careers in medical specialties relative to general practice. It is significant to also note that general practice is sometimes assigned lower status within the medical profession, compared, for example, to surgery and some medical specialties, which may also offer superior pay and more power and influence (e.g.: Alberti et al. 2017; Creed, Searle, and Rogers. 2010; Fazel and Ebmeir 2009).

Based on in‐depth interviews (*n* = 54) with medical students, doctors and educators (*n* = 38), this study explores the cause of these patterns, alongside the consequences for practitioners, the profession and for patients. The broader context is that within the medical profession, these patterns have been broadly welcomed (British Medical Association 2015; Department of Health and Social Care 2018). Historically, areas such as primary care, psychiatry and acute medicine, especially those in ‘under‐doctored’ locations, which are often rural and/or relatively deprived, have been less competitive but also more difficult to fill (Kumwenda et al. 2019). Access to healthcare is an important socioeconomic determinant of health, and where doctors take‐up roles in shortage areas, this contributes to improved service delivery (Dowell et al. 2015). Previous research studies have tended to suggest that these outcomes relate to choice, as, given prior socialisation, doctors from less advantaged backgrounds actively prefer what are sometimes known as ‘prosocial’ careers such as primary care, which are more community orientated and provide opportunities to ‘give back’ (Bennett and Phillips 2010). This has been encouraged as, over the past decade, places have been expanded at existing medical schools and five new ones have been opened, aiming to respond to shortages in areas such as general practice and acute care, and at the same time train more doctors from less advantaged and non‐traditional backgrounds for the profession (Health Education England 2018).

Yet, social stratification could have some less positive effects and these have been relatively overlooked. For example, recent qualitative research studies suggest that structural barriers embedded within education and training raise specific constraints for doctors from less advantaged backgrounds, so that outcomes are sometimes unjust (Ashley and McDonald 2024). An extensive literature exploring occupational segregation also tells us that diversification can be risky for occupations and job roles, as it threatens status (Ashcraft 2013). This deviates from the ‘common‐sense’ position suggesting that status is a ‘natural’ consequence of more complex work found within classical economics (Fevre 1992), and shows that status can be generated using other mechanisms, including as it is ‘borrowed’ from people with which the work is most closely associated (Ashcraft 2013). In most Western industrialised societies, highly educated White men continue to enjoy highest social status overall and, when ‘elite’ occupations diversify from this ‘norm,’ status may dissipate (Cockburn 1981). Because lower status may suggest less influence and power, one implication for medicine is that diversification could make it more difficult for doctors to advocate for the resources they need to deliver safe and effective patientcare. These impacts could, however, be most marked in specialties where doctors from under‐represented or ‘lower’ status backgrounds are most concentrated.

Adopting a critical sociological lens, this article is the first to address these themes. To do so, the specific focus is on how values influence the direction of careers for doctors from less advantaged backgrounds, for what this can tell us about the causes of social stratification and, in turn, how this may relate to the construction and maintenance of hierarchies of status within the profession. The next section describes the conceptual framework used to address these themes.

## 2 Literature Review

### 2.1 Reproduction of (Dis)advantage, Social Stratification and Specialty Status

Arguably the most sophisticated framework to explain mechanisms of social stratification alongside the reproduction of status hierarchies has been provided by French philosopher Bourdieu (e.g. 1984). In brief, Bourdieu conceived of social life as divided between multiple overlapping fields, or spheres of social activity, such as family or profession, which are characterised by a set of informal and formal norms, or rules and logics. Conformity to these rules is played out by actors endowed with field‐appropriate, recognised and convertible forms of capitals, and a more or less appropriately aligned habitus (e.g., Bourdieu 1986). Where capitals are aligned with what is valorised in the field, they offer individuals agency and power. Capitals can be economic (control over financial resources), social (networks of influence and support) and cultural (which exists in three forms: institutionalised through academic qualifications, objectified in cultural goods and embodied, in long‐lasting mental and physical dispositions, such as accent, leisure interests, hobbies and dress). Habitus is a vital concept here, as it reflects these dispositions, suggesting the influence of structure while allowing for individual agency (Brosnan 2009, 56). Habitus comprises patterns of meaning, behaviours and tastes, typically acquired by individuals in early life as they are socialised within the family and at school. In the process, actors are said to internalise expectations and beliefs around what is likely and possible for them, which they reproduce through everyday practices, representing their ‘feel for the game’ in social fields (Edgerton and Roberts 2014, 200).

This framework has been widely used within the sociology of medical education (Brosnan 2009); most recently, Ashley and McDonald (2024) found that the structure of medical education and training sets up barriers and challenges for doctors from less advantaged backgrounds, as they lack access to forms of capital and an appropriately aligned habitus, to help build what is known as ‘portfolio’. To provide brief context for the latter, medical students trained in the UK receive up to 6 years of undergraduate education, which is followed by 2 years of postgraduate training, called ‘Foundation Years’, which can be extended. Doctors may then apply for specialty training. This is either run‐through, for areas such as general practice and paediatrics, or ‘uncoupled,’ the route generally taken by hospital physicians. Access to most specialties has become increasingly competitive in recent years, though historically has been especially intense for some surgical specialties compared to those such as psychiatry, emergency medicine and primary care, which tend to have a strong community focus (NHSE n.d.).

Previous research studies have found that struggles for students from less advantaged backgrounds to access these more competitive specialties can start during education and training. For example, in their review of relevant literature, Krstić et al. (2021) show that medical students from under‐represented backgrounds often lack a sense of belonging, and may experience persistent ‘imposter syndrome’ which can amplify challenges accessing the resources and information necessary to ‘succeed.’ This is particularly the case because, as noted, securing a more competitive specialty position is assisted via extra‐curricular activities which build CV‐points. Activities such as internships and electives, and/or research and conference papers, can be characterised as vital forms of institutionalised cultural capital as they enable distinction relative to peers, but are typically more available to medical students and doctors from more advantaged backgrounds (Ashley and McDonald 2024).

Underlying values also play a role, and a related insight provided by Bourdieu is that ‘success’ tends to require a correspondence between values internalised as habitus and what is valued in the field (Edgerton and Roberts 2014, 197). Previous research studies suggest that doctors from more advantaged backgrounds, especially those with medical family and friends, are more likely to be socialised towards values such as pay and prestige, which map on to more traditional metrics of ‘success’ (Ashley and McDonald 2024). Related advantages may be amplified as they are more likely to share their social background with existing occupational ‘elites,’ who have most power to decide what counts as ‘meritorious,’ and do so in a way which reflects their own traits and skills. While often presented as objective, dispositions which facilitate ‘success’ and conform with the field's *doxa*, or deep structure, can then be quite arbitrary yet critically, appear legitimate as the current social hierarchy is made into an apparent hierarchy of gifts or merit, and naturalised as such (Bourdieu 1997, 2006: see also Edgerton and Roberts 2014, 193). This is an important way in which field structures help defend orthodoxy though, by necessity, their dynamics change, in ways which are significant for related inequalities, as explained next.

### 2.2 Empathy, Emotion Work and Emotional Capital

A key change to medical school cultures and curriculums especially relevant to the current study is that historically doctors were taught early in their training that ‘technical skills are [considered] fundamental, whereas interactive skills (if encouraged at all) are secondary’ (Coulehan and Williams 2003, 9). However, in recent years there has been increasing interest in improving how practitioners relate to patients, as this is said to reduce burnout and have a positive impact on patient satisfaction and distress (Harvey, Stacey, and MacArthur 2023: see also Kerasidou and Horn 2018). Calls for more compassionate care as part of a patient‐centred approach have led to a growing focus on empathy within clinical training, defined as emotional reasoning, allowing physicians to incorporate emotional experiences as part of clinical decision‐making (Vinson and Underman 2020, 1). Previous studies have though noted that discourses of competence and of caring can compete, with the latter often marginalised (Mcleod 2011).

One way we can consider the consequences, for practitioners, patients and the profession, is by extending Bourdieu's forms of capital to include Nowotny's (1981, 148) concept of ‘emotional capital,’ defined as ‘knowledge, contacts, and relations as well as access to emotionally valued skills and assets’. Later conceptualisations drawing from and expanding on the work of Bourdieu made this a specific type of embodied cultural capital, which shapes the development of ‘caring selves’ (Stacey 2011). This concept has something in common with emotional labour (Hochschild 1983), which relates to the work involved in managing one's emotions as they become commodified, so that individual capacities for empathy and warmth are put to corporate use and sold for a wage. However, while emotional labour refers to what people *do*, the concept of emotional capital is more suited to the current research aims, as this can be seen as a *trans*‐situational resource linked to power and privilege, which reproduces the broader structural and cultural conditions in which interactions occur (Cottingham 2016: see also Erickson and Stacey 2013, 179).

From this perspective, and like other forms of embodied cultural capital, emotional capital can be approached as both an *asset,* which is unevenly distributed, and/or a *set of values*, internalised as habitus. As an asset, emotional capital may be associated with emotional resilience and investment in wellbeing, which may or may not be activated in different fields (Lareau and Weininger 2003). As a resource, emotional capital can be seen in more dynamic terms, as the situated frame through which we view the world, guiding our thoughts and actions accordingly (Edgerton and Roberts 2014). In this sense, emotional capital is less a practice, and more an *embodied* attitude towards caring, which includes empathy. To paraphrase Bourdieu (1990, 71), emotional capital ‘learnt by body’ is then not something one *has*, but something one *is*.

Cultural capital of all types is transmitted through the family to drive modes of thinking, types of disposition and sets of meanings and style, suggesting emotional capital is likely to be classed. There is though limited consensus in terms of how. Some scholars suggest that when viewed as an asset, emotional capital is in short supply where working class communities experience humiliation and embarrassment which undermines resilience (Reay 2004). An alternative argument is that related experiences may foster the emotional capital needed to confront adversity (Cottingham 2016). There has been less attention to the classed nature of emotional capital in its more dynamic sense, but some insights are provided by work on emotional labour, again with limited consensus. For example, some scholars suggest children of the middle‐classes are more likely to be exposed to primary socialisation in the family which allows them to grow: ‘sensitive to feeling and [learn] to read it well’ (Hochschild 1983, 158, cited in Payne 2009). However, in their review, Kish‐Gephart et al. (2023) find that social class is *negatively* related to levels of empathy, defined as an individuals' understanding of and sympathy towards others' emotions and affective states. In other words, members of what they term ‘lower’ social classes have been found to show a greater ability to accurately infer other's emotions, while self‐esteem, self‐confidence, self‐efficacy and entitlement have been positively linked to higher social class membership.

These are significant findings given the research study aims here because if we assume students from less advantaged backgrounds are socialised towards values such as empathy and community, this may be positive for their patients yet could be more problematic for them, as practitioners. This may be especially true where technical *competence* is aligned with complexity and considered a skill, whereas *care* is expected to come more ‘naturally,’ and is relatively devalued as has been suggested elsewhere (Quirk et al. 2008). The aim here is to explore these relationships in further depth, for what they reveal about the causes of social stratification, and how this relates to internal status hierarchies within the medical profession. Before expanding on these points, the next section describes the research methods.

## 3 Methods

### 3.1 Sample Group

The sample group for this study comprised medical students, doctors and medical educators (*n* = 38) who were reached in different ways. First, the author undertook a study commissioned by a leading charity, which supports students from less advantaged socioeconomic backgrounds to access medical school. Eligibility for the programme is determined by having been in receipt of Free School Meals at age 14, which is indicative of a family income significantly below the national average, and is also how ‘less advantage’ is defined here. Alumni of the programme were first invited to participate in this research study in 2019, with the purpose of exploring their experiences in education and training and, where relevant, their journey into speciality careers. At this point thirty (*n* = 30) agreed to participate who were either studying or had graduated from a total of 14 medical schools across the UK. Having given their informed consent, these students were contacted again in 2023, and just over half (*n* = 16) agreed to participate in a follow‐up interview. During this second phase of the project, the research was extended to include contextual interviews with more experienced clinicians and medical educators (*n* = 8), purposively selected by the author from existing networks, as they had professional experience of the widening participation agenda. These participants also made introductions to colleagues and peers with relevant knowledge and experience. Socioeconomic background was not part of the eligibility criteria for this second sample group, but all had markers of less advantage, as first‐in‐family to attend university and/or parents in routine or semi‐skilled labour. Ethical approval for this study was granted by the author's institution (reference QMERC22.352). Further details of the sample group and demographics are provided in Table 1.

**TABLE 1:** Participant demographics.

Pseudonym	Sex	Ethnicity	Job role	When interviewed (stage of education/career)
Amani	F	Bangladeshi	Medical student	2019 (UG2); 2023 (UG5)
Amal	F	Arab	Doctor	2019 (FY1); 2023 (FY4)
Amelia	F	Black African	Medical student	2019 (UG2); 2023 (UG5)
Anya	F	Indian	Medical student	2019 (UG2); 2023 (UG5)
Arun	M	Asian	Doctor	2019 (ST1)
Ben	M	White British	Medical student	FY2
Brian	M	White British	Doctor	2023 (GP)
Damian	M	White British	Medical student	2019 (UG2); 2023 (UG5)
Devaaj	M	Asian	Doctor	2023 (GP)
Ella	F	White British	Medical student	2019 (UG1); 2023 (UG4)
Eva	F	Mixed Race	Doctor	2019 (UG4); 2023 (FY1)
Flora	F	White British	Medical student	2019 (UG3)
James	M	White British	Medical student	2019 (UG2)
Joe	M	White British	Medical student	2019 (UG2)
Fatima	F	Pakistani	Doctor	2019 (FY3)
Huma	F	Pakistani	Doctor	2019 (UG5); 2023 (FY1)
Jamila	F	Arab	Medical student	2019 (UG3)
Kathryn	F	White British	Medical educator	2023 (n/a)
Lucy	F	Bangladeshi	Medical student	2019 (UG2); 2023 (UG4)
Leon	M	Black African	Medical student	2019 (UG5)
Maarisa	F	Pakistani	Medical student	2019 (UG3)
Malik	M	Arab	Medical student	2019 (UG3)
Matilda	F	White British	Doctor	2023 (consultant)
Nabila	F	Bangladeshi	Medical student	2019 (UG3); 2023 (UG5)
Nigel	M	Mixed Race	Medical student	2019 (UG2); 2023 (UG5)
Nicola	F	White British	Medical student	2019 (UG1)
Omari	M	Black African	Doctor	2019 (FY1)
Rashid	M	Arab	Doctor	2019 (FY1); 2023 (ST1)
Richard	M	White British	Medical educator	2023
Reena	F	Pakistani	Medical student	2019 (UG2)
Sally	F	White British	Doctor	2023 (GP)
Sue	F	White British	Medical educator	2023 (n/a)
Sureisha	F	British Asian	Medical student	2019 (UG2); 2023 (UG5)
Seren	F	Indian	Medical student	2019 (UG2); 2023 (UG5)
Thanh	M	British Asian	Medical student	2019 (UG2)
Ursula	F	White European	Doctor	2019 (ST1); 2023 (ST4)
Zara	F	Pakistani	Medical student	2019 (UG2)
Zoe	F	White European	Doctor	2019 (FY1); 2023 (ST1)

### 3.2 Data Collection

All interviews were conducted by the author on the basis of informed consent, confidentiality and anonymity (during the first phase of the research in 2019/20, interviews were conducted by telephone and in the second phase, online using Microsoft Teams). Interviews took between 60 and 90 minutes, and all were recorded and transcribed, accompanied by field notes. Transcripts were uploaded to the qualitative data software package Nvivo (v12), and identifying details of participants were removed. Participants were asked how they define a ‘good’ doctor and how they define a ’successful’ doctor, the extent to which they felt these definitions overlap and what characteristics and practices they associated with both. They were also asked what they thought is necessary to ‘get ahead’ in medicine, along with how (and when) they had learnt this information, and whether their understanding had changed over time. Where relevant, participants were also asked why they chose to study medicine, about their experiences during education and training, what they most valued as they looked ahead and/or pursued their medical career, and whether this had changed.

### 3.3 Data Analysis

Data analysis was arranged around two research questions. First, what do participants believe is most *valuable* when it comes to building medical careers: in other words, what traits, characteristics, attitudes and skills have most worth, especially to secure ‘success?’ Second, what do participants most *value* as they pursue their medical career, in terms of what is important to them? These questions were informed by Bourdieu's conceptual framework, and relate to the structure of the field of medical education and training, and to individual portfolios of capital and habitus, respectively. The over‐arching objective was to explain both the causes of social stratification and related inequalities, and how this might reflect and reproduce status hierarchies within the profession.

Data analysis used a thematic approach, starting with a close reading of all transcripts, performed by the author, to identify sections where participants addressed subjects relating to the two research questions, after which extracts under each heading were re‐read and coded, working iteratively between theory and data.

With respect to the first question, the focus was especially on the rules and logics participants believe define the field, and forms of capital they felt most convertible to symbolic capital, in the form of status, reputation and respect. Medicine is of course a highly differentiated career, comprising overlapping fields. As such, it is unlikely that one set of rules and logics reflects what is valuable across this complex social space, as the findings show. With respect to the second question, the focus was on values internalised as individual habitus. Here, close reading of the data revealed most participants entered the field better aligned with characteristics they associated with the ‘good’ doctor. Given the dynamic nature of capital and habitus, it is of course possible to distinguish between capital gained in primary socialisation, typically within family and the home, and capital gained in secondary socialisation, including in higher education and occupation (Bourdieu and Wacquant 1992). However, analysis suggested that participants' values remained relatively stable over time, even as some became more aware of a mismatch between those internalised as habitus and what is valued in the field. In what follows, findings are presented under two headings, corresponding with the two research questions.

## 4 Findings

### 4.1 Learning What Is Valuable in Medical Careers

Participants explained that as they had started medical school, and looked ahead to education and training, they had believed that along with an aptitude for science and an interest in people, relevant knowledge would be most valuable in the field, as an indication of the potential to become a safe and competent doctor. In turn, they expected that the acquisition of knowledge would be more available for students who had internalised the capacity for persistence and hard work, contributing to high academic attainment. For example, as Arun said: ‘I knew that knowledge would be everything in medicine, what's the point of a doctor that doesn't know anything?…that takes hard work.’ Participants described how once they arrived at medical school these expectations were initially confirmed within the formal curriculum, where they were regularly reminded by medical educators to concentrate on exam success. Eva explained: ‘The message from our teachers was to focus on exams, to work hard and do the best we can, because that's how we prove we're competent, and that's what matters most in medicine.’

These skills, traits and characteristics are arguably consistent with more traditional notions of medical professionalism, where possession of knowledge suggests competency and effective practice (e.g.: Kerasidou and Horn 2018). However, participants explained how modules on clinical practice during the first one or two undergraduate years also reinforced the value of empathy and compassionate care. James described, for example, how he and his peers were regularly reminded that good doctors are: ‘empathetic and sympathetic about the patient that they're dealing with.’ Amelia described learning that a good doctor is: ‘someone that listens to other people, can work well in a team setting and communicates well, because we've been doing some work about how the team works and how important it is, and they start working on us building our communication skills right from the beginning.’ Ella said: ‘You could really tell [medical educators], they want you to soak up that thing of listening to your patients, to understand where they are coming from, not just being the arrogant doctor who just tells them what to think.’ The need for empathy could extend to colleagues, as participants also explained how they were encouraged within the formal curriculum to prioritise teamwork, effective communication and collaboration. As Joe said: ‘Doctors don't work alone, they work in teams and one of the other things we've been taught is communication skills, so we can work together and get along.’

This focus on clinical skills might be seen as an important aspect of what Bourdieu called secondary socialisation, consistent with the more recent emphasis on developing empathy during medical education (e.g.: Harvey, Stacey, and MacArthur 2023; Vinson and Underman 2020). Previous literature has pointed to some tensions here, finding that the accumulation of knowledge is not always conducive to the expression of emotion and that doctors may sacrifice emotional wellbeing, their own or their patients', to meet the demands of their chosen career paths (Underman 2015). To a large extent, as they embarked on education and training, participants seemed prepared for this tension, as again, Joe explained: ‘I do think it's totally possible to be really knowledgeable and really good at what you do and still really care about your patients…I think I can do that. I hope I can.’ However, as they entered clinical placements, participants started to realise that a different set of traits and skills may be valuable, especially when it comes to securing a more competitive career. For example, during her early years at medical school, Eva said: ‘It did feel really collaborative. Like, we're all in it together.’ During her Foundation Years, she revised her opinion to now believe: ‘It's totally dog eat dog.’ Nabila made some similar points:

It’s actually not at all collaborative…I mean sometimes it is, like a friend might tell you when there’s an opportunity to do some research, or something like that. Or I’ve had people tell me what I need to do, like go to conferences, that kind of thing. But you know, when it comes to building portfolio it’s about accumulating points, more than other people have…so you’re kind of out for yourself, especially if you’re ambitious.

Awareness of these requirements generally developed as students encountered more senior clinicians, and more experienced medical students, with whom they engaged in informal interactions, representing part of the ‘hidden curriculum.’ This is a key mechanism through which medical students are socialised into professional norms (e.g.: Hafferty and Franks 1994; MacNeil, Regehr, and Holmes 2022), and refers to rules and regulations that are not explicit or codified (Bourdieu and Wacquant 1992). While most participants felt fairly confident that expertise and empathy could co‐exist, some hinted that the latter might be difficult to reconcile with the individualistic approach required to secure traditional measures of ‘success.’ Related tensions were highlighted by Sally, a medical educator and senior doctor, who explained how characteristics such as empathy may be suppressed even before students reach medical school: ‘You know, these very, very competitive people fighting their way to the top of the pile…perhaps lose some of the empathy.’ Fatima suggested these tensions could persist into later carers as getting noticed by more senior clinicians, and engaging in the intense competition required to build an impressive portfolio, may work against this more compassionate and collaborative approach:

I definitely don’t want to say that people in those really competitive jobs don’t have empathy, they definitely do…but I don’t know how easy it is to be really caring when you’re always fighting to secure the best opportunities…to get those sort of top jobs, empathy is sort of a nice to have, whereas all that stuff that goes into a portfolio, that’s a must have.

However, it is not only that empathy and emotional capital are less consistent with the practices and attitudes required to access more competitive roles but that their expression may be actively *devalued*. On this point, participants tacitly agreed that a more compassionate approach is not only more obviously aligned with less competitive careers but may also confirm their construction as such, as Omari explained:

I think geriatrics or maybe palliative care are examples where, you’re dealing with the patients but also with their family and there is that community focus, you really need to show you care. Maybe you’re dealing with end‐of‐life…those skills are so, so important to patients but I don’t think they always sit that well with perceptions of, you know, the ‘big expert’…[empathy] is not that valuable to get the best jobs…academic medicine is really competitive but it’s not important there.

Huma explained how she was advised to balance expressions of empathy with a more detached approach. ‘I’ve been warned on hospital rotations,’ she said, ‘to be careful not to look too caring because people will think I’m a nurse.’ Huma illustrates here how socioeconomic background intersects with gender as the role of ‘nurse’ is coded as female, and associated with care, in ways apparently interpreted by some practitioners as indicative of lower skill. These associations might also be explained as academic attainment remains very highly valued in medicine as a form of institutionalised cultural capital. In contrast, emotional capital is less easily captured by traditional forms of certification and is considered something most doctors can do, as Ursula explained: ‘We know empathy is really important, I really believe the best doctors are the ones who listen to their patients, they’re compassionate … [but] it’s not something you put on your CV, it’s not going to make you stand out.’ A related implication is that emotional capital is less convertible to other capitals valorised in the field, which confer power and agency, along with reputation and ‘respect.’

### 4.2 What do Participants Value as They Pursue Their Careers?

Having explored what participants consider valuable in the field of medical education and training, the focus of this section is on what they value as they build their career. While there is clearly variation here, participants generally felt more closely aligned with traits they believe characterise a ‘good’ doctor and experience less congruence with those associated with the ‘successful’ doctor. This should not imply more competitive careers are never attractive to doctors from less advantaged backgrounds or beyond their reach. It does though help to explain why the majority of participants aspired towards or were training in more community orientated careers, such as psychiatry, acute medicine, and primary care, preferences which appear to relate to early socialisation.

We can start here by considering why participants chose medicine and what they wanted and expected from a medical career. Most described an aptitude for science, along with an interest in people, as is common amongst aspirant medics. So too is a family history in medicine, and because participants were first‐in‐family to attend university, the latter could not apply to them. Nevertheless early encounters with the profession during their childhood were highly significant.

Over half of the participants in this research study described having spent time with doctors when they were young as a result of their own or family members' ill‐health. This made medicine more familiar and thus imaginable but also seemed to engender a particular attitude towards caring, empathy and close patient support which can be understood as a ‘predisposition, tendency, propensity, or inclination and internalised as habitus’ (Bourdieu 1977, 213). For example, Nicola’s father had died when she was a teenager and, she explained, her motivation towards medicine: ‘all came from that ‐ the classic, like being able to help people.’ Damian’s sister had received in‐patient psychiatric care which he found inadequate. He wanted to: ‘see if I could do any better…to see if I could help some people…see what kind of difference I could make.’ This sense of having been disappointed by the medical profession could also relate to participants feeling they and/or their community had been patronised or misunderstood, often particularly highlighting how socioeconomic background intersects with ethnicity. As Fatima said: ‘I always felt growing up, like there was this lack of understanding…partly that’s to do with language maybe, like, literally…but there was also this feeling that doctors just didn’t understand, I don’t know, the struggles we had…studying medicine was a way to make up for that’.

It is entirely possible that students from more advantaged backgrounds enter medical school with similar sensitivities. However, the types of experience outlined above may be particularly common for students from less advantaged backgrounds because socioeconomic disadvantage is associated with a wide range of mental and physical health conditions, both chronic and acute. These prior experiences indicate how socialisation may particularly orientate medical students from less advantaged backgrounds towards empathy and related forms of emotional capital as both a core value and an internalised attitude to care well before medical school. An additional indication of such is that while by no means the only factor directing their careers, a matter of central importance to many participants was how they could be of most value to their community. As Amani said: ‘Medics from low socioeconomic backgrounds, we know the struggles…giving back to our community is a big component of our personalities’. Participants often compared themselves to doctors from more advantaged backgrounds in this respect, who they felt might enter education and training more likely to embody confidence and even entitlement as a key form of cultural capital, and more closely aligned with specialities in which these traits are most valuable. Some also felt these more advantaged peers could lack empathy towards diverse communities, as Amal said:

We were having a lecture on what to do with patients with limited English…I heard [another student] say, ‘oh well, she shouldn’t be here with that unga bunga language’…people from [more privileged] backgrounds, they’ve not had much exposure and that really impacts people in lower socio‐economic groups…it really changes how they approach care…it doesn’t mean they can’t still be very successful though.

As Amal hints, lacking sensitivity towards difference is by no means a barrier to a ‘successful’ career. However, it is equally important to underline that a drive to ‘give back’ can be expressed in many different specialities and a related caveat is that when related attitudes and beliefs are internalised as habitus, this does not rule out more competitive careers. Some participants chose to enact related values, fuelled by a range of factors including personality and experiences of adversity. As one example, Omari was working towards a highly competitive surgical speciality when he took part in this research study. He said he had adopted the requisite competitive and individualistic stance as the prospect of a medical career, and surgery in particular, represented a means to escape the precarious conditions of his past. ‘As a kid from low socioeconomic status,’ he explained, ‘it will feel like you've been given this golden ticket to leave this life…you're willing to fight tooth and nail to make sure you keep that ticket.’

Overall, though, it was more common for participants in this research study to actively reject more extreme forms of individualism and overt competition, as inauthentic for them. Echoing points introduced earlier on, a strong motivation for Maarisa to train as a doctor was because her father had been unwell during her childhood. ‘For me,’ she said, ‘it was the empathy side of it…the human aspect…what it’s like for patients who have chronic diseases because that was the example that my dad had.’ For her, fighting to access a more prestigious or competitive speciality was not a priority. ‘It’s not who I am’, she said, ‘to get into a [more competitive] career, you have to play the competitive game…for me personally, I just don’t want to do that.’

These examples offer further insights into how values internalised as habitus might translate into socially stratified careers, orientating students and doctors from less advantaged backgrounds towards prosocial, or community‐focused areas, in relatively high numbers. Critically, participants often framed these outcomes as an expression of agency and as such, in a positive light, especially as this allowed them to prioritise a more fulfiling career over values such as prestige or pay, as Ella explained: ‘Maybe if I was from a [privileged] background…there's more pressure for high achieving prestige… my parents are just, you know, whatever you do, be happy.’ However, we can also note Bourdieu's (1977, 77) belief that social actors make a virtue out of necessity, as social structures and external forces influence their understanding of what seems reasonable and practicable in a given situation. It is useful here to consider the role emotional capital might play as a specific *resource*, in the form of resilience. A consistent theme raised by participants was that while medical education is challenging for everyone, the structure of the field presents particular challenges for those from under‐represented and less advantaged backgrounds. For example, limited economic capital contributed to persistent financial concerns, leaving some participants feeling isolated from peers and less able to participate in the life of their medical school, as Nicola explained:

It’s always been a constant worry in the back of my head that I have to be aware of finances, I could run out of money. And then just the whole thing of getting involved in the medical school community and culture…like, all your friends are doing a holiday here, and you can’t join them…I have at times had to be more isolated from the rest.

Participants also described how regular micro‐aggressions at medical school and during training drew attention to their embodied cultural capital and generated a sense they lacked ‘fit.’ According to Amani:

You have to be confident about yourself, as well, because people are going to say different things about you and put you down, say, ‘oh, how can you ever be a doctor?’…if you speak in a certain way…[it] will then convert to, ‘oh, he's from that dirty area, that gang area’…Or people from Essex…[it will be] ‘oh, this person is from Essex. He's dumb’.

On average, doctors from more advantaged backgrounds who are less likely to experience these challenges may also have more emotional bandwidth with which to engage in the most competitive behaviours. In contrast, as participants often described struggling with their mental health as they battled feelings they did not ‘belong,’ their appetite for a more competitive game, as the *affective* component of habitus, was often reduced. As Flora said: ‘Medical school is really hard and then there's life…I've been supporting my family…so now my goal is just to get out with my mental health intact.’ As such, while this study underlines that values matter in directing speciality careers, the findings reported here also suggest caution should be applied when suggesting a natural or inevitable association between ‘lower’ class of origin and less competitive areas, and/or prosocial specialities. As Seren explained: ‘[Working class doctors] are not here to fill a gap, we are all whole individual human beings with our own hopes and desires.’

## 5 Discussion

The aim of this paper has been to consider the causes of social stratification, how this maps onto and helps inform speciality status and what might be the consequences for patients, practitioners and the medical profession. Guided by core concepts provided by Bourdieu (e.g. 1984), this study found first that emotional capital and empathy are valued by participants, whose prior socialisation means they have often internalised related values as an attitude towards care, suggesting a high level of ‘fit’ with more prosocial careers such as primary care. However, they argued empathy is not always considered valuable throughout the profession, especially in more competitive careers, as it is also associated with less skill, status and prestige. As has been said elsewhere: ‘Embodied feelings emerge from one's position within social hierarchies at the same time that emotional practices help to maintain those hierarchies’ (Cottingham, Johnson, and Erickson 2018, 145). Building from this point, the core argument here is that social stratification and hierarchies of speciality status are closely related, and should be recognised as such, both in theory and in practice.

Before expanding on the implications for various stakeholder groups, it is important to note certain limitations of this study, one of which is that it was relatively small scale. Research exploring the causes of social stratification using a larger sample group could help us understand the dispositions of doctors from a range of backgrounds, to further pinpoint the effects of socioeconomic background on speciality decision. Statistical research could usefully explore the relationship between competition ratios to specialities (as a proxy for status) and demographics on entry, to provide further insights into how these relate, and changes over time.

Other qualifications and caveats include that the argument presented here should not imply some sort of binary between doctors from more and less advantaged backgrounds, whose stocks of ‘emotional capital’ are fundamentally and universally different. Neither should it suggest that an orientation towards a less competitive or community based speciality is a bad or negative choice. Instead, we underline that an association with lower skill means these outcomes may sometimes be *constructed* as such. Currently, this may not seem a pressing concern given that the UK medical profession remains extremely homogenous in demographic terms, yet the profession is likely to continue to diversify. Given intense competition and intra‐professional conflict, as medical practitioners struggle for resources it is possible that social stratification may intensify.

Against this backdrop, the current study points towards several related challenges. One is that where a commitment to more community orientated careers is framed as a particular vocation, this could make doctors in these roles more available to exploitation, even to become the profession's ‘emotional proletariat.’ The latter term was used by Bolton (2004, 33) to describe the ‘lowest order of emotion workers.’ In the current context, it is used to describe doctors working in areas which require especially high expenditure of emotional capital as an attitude towards caring and as a form of emotional resilience.

It is important to underline that most doctors face these risks, especially as burnout is becoming more prevalent in UK medicine (GMC 2023). However, these demands may be especially intense in specialties experiencing shortages and in under‐doctored areas, where some evidence suggests doctors also receive less support (Brewster, Lambert, and Shelton. 2022). The profession's regulatory body, the General Medical Council (GMC), conducts a National Training Survey (2023), which offers the related insight that the specialty where trainees are most susceptible to burnout is emergency medicine. This specialty also experiences amongst the most acute shortages and was amongst the most popular career destinations for participants in this research study. These relationships raise concerns with social justice and patient care, including as burnout is associated with lower rates of retention, a problem with which the profession is already struggling. Understanding these relationships would benefit from further research, though in the meantime, this article concludes by considering how medical educators and policy makers might respond.

## 6 Conclusion

In the context of a growing professional emphasis on clinical empathy, Harvey, Stacey, and MacArthur (2023, 6) have pointed to an ‘elephant in the room:’ namely, that enacting empathy comes with multiple benefits for patients and even health systems but sometimes at the expense of providers, especially where expending emotional capital is not supported, valued or remunerated. One implication is that stakeholders across the profession should continue existing work to revalue emotional capital by challenging established norms (while continuing to recognise intellectual capital and technical skill and recognising that the two can co‐exist) (Crossman 2024). This might require making efforts to ensure steep status hierarchies between specialties are less likely to exist.

Whether this is possible or indeed universally attractive across the profession is though uncertain because related hierarchies and a degree of stratification may support the interests of those in higher status jobs. To understand these themes, we can refer to Weberian analyses, which consider how occupational elites defend status and prestige using occupational closure mechanisms. These ensure non‐eligible groups are kept out to create an artificial illusion of scarcity in available skills and an exclusive image, though this takes place on apparently meritocratic and thus legitimate grounds (Bolton and Muzio 2007). Historically, this has been achieved by restricting the supply of labour using formal credentials though, nowadays, informal mechanisms have become equally important, often using forms of embodied cultural capital as signs of suitability, such as accent, dress and leisure interests (Ashley and McDonald 2024). Occupational closure mechanisms are of course subtle and rarely exercised with entirely conscious intent. However, as the medical profession struggles with declining working conditions, we might expect that *internal* closure will become more intense, as those at the ‘top’ struggle to protect their existing privileges, and one way they can do so is by keeping ‘others’ out.

We can remember here that: ‘the value attached to different forms of capital are stakes in the struggle between different class fractions’ (Reay 2004, 58). Relatedly, non‐certified social skills such as empathy rarely attract the highest prestige or indeed pay (Payne 2009, 361). These are circumstances in which empathy and emotional capital could become ways to help ensure existing hierarchies are kept in place. A profession which is polarised in this way is though likely to have invidious effects, including to undermine effective communication between doctors in different specialties. While medicine represents a complex set of overlapping fields, this article ends by advocating for a collaborative approach to tackle the challenge of social stratification, to help protect patients, practitioners and the profession, at a point when arguably, its power and status are under attack (Nettleton 2021).

## Author Contributions

**Louise Ashley:** conceptualisation, formal analysis, methodology, project administration, investigation, writing–original draft, writing–review and editing.

## Ethics Statement

This project was granted ethics approval by QMUL's Research Ethics Committee, reference QMERC22.352).

## Consent

The author has nothing to report.

## Conflicts of Interest

The author declares no conflicts of interest.

## Permission to Reproduce Material From Other Sources

The author has nothing to report.

## Data Availability

The data supporting the findings are stored by the author under secure conditions. The data are not publicly available as they contain information that would compromise the privacy and confidentiality of research participants. Further, the latter took part in the project on the basis of informed consent, which did not make provision for their data to be shared publicly in full.

## References

[shil13860-bib-0001] Alberti, H. , K., Banner , H. Collingwood , and K. Merritt . 2017. “Just a GP’: A Mixed Method Study of Undermining of General Practice as a Career Choice in the UK.” British Medical Journal Open 7, no. 11: e018520. 10.1136/bmjopen-2017-018520. PMC5722080 29102997

[shil13860-bib-0002] Ashcraft, K. L. 2013. “The Glass Slipper: ‘Incorporating’ Occupational Identity in Management Studies.” Academy of Management Review 38, no. 1: 6–31. 10.5465/amr.2010.0219.

[shil13860-bib-0003] Ashley, L. , and I. McDonald . 2024. “When the Penny Drops: Understanding How Social Class Influences Speciality Careers in the UK Medical Profession.” Social Science and Medicine 348: 116747. 10.1016/j.socscimed.2024.116747. 38547804

[shil13860-bib-0004] Bennett, K. L. , and J. P. Phillips . 2010. “Finding, Recruiting, and Sustaining the Future Primary Care Physician Workforce: A New Theoretical Model of Specialty Choice Process.” Academic Medicine 85, no. 10: S81–S88. 10.1097/acm.0b013e3181ed4bae. 20881711

[shil13860-bib-0005] Bolton, S. C. 2004. “A Simple Matter of Control? NHS Hospital Nurses and New Management.” Journal of Management Studies 41, no. 2: 317–333. 10.1111/j.1467-6486.2004.00434.x.

[shil13860-bib-0006] Bolton, S. C. , and D. Muzio . 2007. “Can't Live with'Em; Can't Live Without'em: Gendered Segmentation in the Legal Profession.” Sociology 41, no. 1: 47–64. 10.1177/0038038507072283.

[shil13860-bib-0007] Bourdieu, P. 1977. Outline of a Theory of Practice. Cambridge: Cambridge University Press.

[shil13860-bib-0008] Bourdieu, P. 1984. Distinction: A Social Critique of the Judgement of Taste. Harvard: Routledge and Kagan Paul Ltd.

[shil13860-bib-0009] Bourdieu, P. 1986. “The Struggle for Symbolic Order.” Theory, Culture and Society 3, no. 3: 35–51.

[shil13860-bib-0010] Bourdieu, P. 1990. The Logic of Practice. Cambridge: Polity Press.

[shil13860-bib-0011] Bourdieu, P. 1997. “The Forms of Capital.” In Education: Culture, Economy, Society, edited by A. H. Halsey , H. Lauder , P. Brown , et al., 46–58. Oxford: Oxford University Press.

[shil13860-bib-0012] Bourdieu, P. 2006. “Cultural Reproduction and Social Reproduction.” In Inequality: Classic Readings in Race, Class, and Gender, edited by D. B. Grusky and S. Szelényi , 257–271. Boulder, CO: Westview Press.

[shil13860-bib-0013] Bourdieu, P. , and L. Wacquant . 1992. An Invitation to Reflexive Sociology. Chicago, Il: University of Chicago Press.

[shil13860-bib-0014] Brewster, L. , M. Lambert , and C. Shelton . 2022. “Who Cares where the Doctors Are? The Expectation of Mobility and its Effect on Health Outcomes.” Sociology of Health & Illness 44, no. 7: 1077–1093. 10.1111/1467-9566.13480. 35583963 PMC9545762

[shil13860-bib-0015] British Medical Association . 2015. The Right Mix: How the Medical Profession Is Diversifying its Workforce. London: BMA.

[shil13860-bib-0016] Brosnan, C. 2009. “Pierre Bourdieu and the Theory of Medical Education: Thinking ‘Relationally’ About Medical Students and Medical Curricula.” In Handbook of the Sociology of Medical Education, 51–68. London: Routledge.

[shil13860-bib-0017] Cockburn, C. 1981. “The Material of Male Power.” Feminist Review 9, no. 1: 41–58. 10.1057/fr.1981.19.

[shil13860-bib-0018] Cottingham, M. D. 2016. “Theorizing Emotional Capital.” Theory and Society 45, no. 5: 451–470. 10.1007/s11186-016-9278-7.

[shil13860-bib-0019] Cottingham, M. D. , A. H. Johnson , and R. J. Erickson . 2018. “‘I Can Never Be Too Comfortable’: Race, Gender, and Emotion at the Hospital Bedside.” Qualitative Health Research 28, no. 1: 145–158. 10.1177/1049732317737980. 29094641 PMC5714163

[shil13860-bib-0020] Coulehan, J. , and P. C. Williams . 2003. “Conflicting Professional Values in Medical Education.” Cambridge Quarterly of Healthcare Ethics 12, no. 1: 7–20. 10.1017/s0963180103121032. 12625198

[shil13860-bib-0021] Creed, P. A. , J. Searle , and M. E. Rogers . 2010. “Medical Specialty Prestige and Lifestyle Preferences for Medical Students.” Social Science and Medicine 71, no. 6: 1084–1088. 10.1016/j.socscimed.2010.06.027. 20674118

[shil13860-bib-0022] Crossman, S. 2024. Why the Emotional Labour of Hospital Staff Is Dirty Work. Aeon Essays. https://aeon.co/essays/why‐the‐emotional‐labour‐of‐hospital‐staff‐is‐dirty‐work

[shil13860-bib-0023] Department of Health & Social Care . 2018. A Mandate from the Government to Health Education England. London: Crown Office.

[shil13860-bib-0024] Dowell, J. , M. Norbury , K. Steven , and B. Guthrie . 2015. “Widening Access to Medicine May Improve General Practitioner Recruitment in Deprived and Rural Communities: Survey of GP Origins and Current Place of Work.” BMC Medical Education 15: 1–7. 10.1186/s12909-015-0445-8. 26428081 PMC4591588

[shil13860-bib-0025] Edgerton, J. D. , and L. W. Roberts . 2014. “Cultural Capital or Habitus? Bourdieu and Beyond in the Explanation of Enduring Educational Inequality.” Theory and Research in Education 12, no. 2: 193–220. 10.1177/1477878514530231.

[shil13860-bib-0026] Erickson, R. J. , and C. L. Stacey . 2013. “Attending to Mind and Body: Engaging the Complexity of Emotion Practice Among Caring Professionals.” In Emotional Labor in the 21st Century, 175–196. London: Routledge.

[shil13860-bib-0027] Fazel, S. , and K. P. Ebmeier . 2009. “Specialty Choice in UK Junior Doctors: Is Psychiatry the Least Popular Specialty for UK and International Medical Graduates?” BMC Medical Education 9, no. 1: 1–7. 10.1186/1472-6920-9-77. 20034389 PMC2805648

[shil13860-bib-0028] Fevre, R. 1992. The Sociology of Labour Markets. London: Harvester Wheatsheaf.

[shil13860-bib-0029] Friedman, S. , Laurison, D. and Macmillan, L. 2017. Social Mobility, the Class Pay Gap and Intergenerational Worklessness: New Insights From the Labour Force Survey. Social Mobility Commission. https://assets.publishing.service.gov.uk/government/uploads/system/uploads/attachment_data/file/596945/The_class_pay_gap_and_intergenerational_worklessness.pdf

[shil13860-bib-0030] General Medical Council . 2023. National Training Survey. https://www.gmc‐uk.org/education/how‐we‐quality‐assure‐medical‐education‐and‐training/evidence‐data‐and‐intelligence/national‐training‐surveys.

[shil13860-bib-0031] Hafferty, F. W. , and R. Franks . 1994. “The Hidden Curriculum, Ethics Teaching, and the Structure of Medical Education.” Academic Medicine 69, no. 11: 861–871. 10.1097/00001888-199411000-00001. 7945681

[shil13860-bib-0032] Harvey, S. D. , C. L. Stacey , and K. R. MacArthur . 2023. “The Empathic Capital of Pre‐Medical Students.” SSM‐Qualitative Research in Health 3: 100236. 10.1016/j.ssmqr.2023.100236.

[shil13860-bib-0033] Health Education England . 2018. New Medical Schools to Train Doctors of the Future. London: HEE.

[shil13860-bib-0034] Hochschild, A. 1983. The Managed Heart: Commercialization of Human Feeling. Berkeley: University of California Press.

[shil13860-bib-0035] Kerasidou, A. , and R. Horn . 2018. “Empathy in Healthcare: The Limits and Scope of Empathy in Public and Private Systems.” In Marketisation, Ethics and Healthcare, 163–173. London: Routledge.

[shil13860-bib-0036] Kish‐Gephart, J. J. , K. J. Moergen , J. D. Tilton , and B. Gray . 2023. “Social Class and Work: A Review and Organizing Framework.” Journal of Management 49, no. 1: 509–565. 10.1177/01492063221076822.

[shil13860-bib-0037] Krstić, C. , L. Krstić , A. Tulloch , S. Agius , A. Warren , and G. A. Doody . 2021. “The Experience of Widening Participation Students in Undergraduate Medical Education in the UK: A Qualitative Systematic Review.” Medical Teacher 43, no. 9: 1044–1053. 10.1080/0142159x.2021.1908976. 33861176

[shil13860-bib-0038] Kumwenda, B. , J. Cleland , G. Prescott , K. Walker , and P. Johnston . 2019. “Relationship Between Sociodemographic Factors and Specialty Destination of UK Trainee Doctors: A National Cohort Study.” BMJ Open 9, no. 3: e026961. 10.1136/bmjopen-2018-026961. PMC6475150 30918038

[shil13860-bib-0039] Lareau, A. , and E. B. Weininger . 2003. “Cultural Capital in Educational Research: A Critical Assessment.” Theory and Society 32, no. 5/6: 567–606. 10.1023/b:ryso.0000004951.04408.b0.

[shil13860-bib-0040] MacLeod, A. 2011. “Caring, Competence and Professional Identities in Medical Education.” Advances in Health Sciences Education 16, no. 3: 375–394. 10.1007/s10459-010-9269-9. 21188513

[shil13860-bib-0041] MacNeil, K. A. , G. Regehr , and C. L. Holmes . 2022. “Contributing to the Hidden Curriculum: Exploring the Role of Residents and Newly Graduated Physicians.” Advances in Health Sciences Education 27, no. 1: 201–213. 10.1007/s10459-021-10081-8. 34822055

[shil13860-bib-0042] Medical Schools Council . 2018. Selection Alliance 2018 Report: An Update on the Medical Schools Council’s Work in Selection and Widening Participation. London: MSC.

[shil13860-bib-0043] Nettleton, S. 2021. The Sociology of Health and Illness. Cambridge: Polity Press.

[shil13860-bib-0044] NHS England . https://medical‐heenhs‐uk/medical‐trainining‐recruitment/competition‐ratios/2022‐competition‐ratios. Accessed 9th October 2023.

[shil13860-bib-0045] Nowotny, H. 1981. “Austria: Women in Public Life.” In Access to Power: Cross‐National Studies of Women and Elites, edited by C. F. Epstein and R. L. Coser , 147–156. London: George Allen & Unwin.

[shil13860-bib-0046] Payne, J. 2009. “Emotional Labour and Skill: A Reappraisal.” Gender, Work and Organization 16, no. 3: 348–367. 10.1111/j.1468-0432.2009.00448.x.

[shil13860-bib-0047] Quirk, M. , K. Mazor , H. L. Haley , et al. 2008. “How Patients Perceive a Doctor's Caring Attitude.” Patient Education and Counselling 72, no. 3: 359–366. 10.1016/j.pec.2008.05.022. 18684582

[shil13860-bib-0048] Reay, D. 2004. “It's All Becoming a Habitus’: Beyond the Habitual Use of Habitus in Educational Research.” British Journal of Sociology of Education 25, no. 4: 431–444. 10.1080/0142569042000236934.

[shil13860-bib-0049] Santana, I. R. , and M. Chalkley . 2017. “Getting the Right Balance? A Mixed Logit Analysis of the Relationship Between UK Training Doctors’ Characteristics and Their Specialties Using the 2013 National Training Survey.” British Medical Journal Open 7, no. 8: e015219. 10.1136/bmjopen-2016-015219. PMC5724110 28801397

[shil13860-bib-0050] Stacey, C. L. 2011. The Caring Self: The Work Experiences of Home Care Aides. Cornell: Cornell University Press.

[shil13860-bib-0051] The National Training Survey . 2023. Initial Findings Report. London: GMC.

[shil13860-bib-0052] Underman, K. 2015. “Playing Doctor: Simulation in Medical School as Affective Practice.” Social Science and Medicine 136: 180–188. 10.1016/j.socscimed.2015.05.028. 26022187

[shil13860-bib-0053] Vinson, A. H. , and K. Underman . 2020. “Clinical Empathy as Emotional Labor in Medical Work.” Social Science and Medicine 251: 112904. 10.1016/j.socscimed.2020.112904. 32151886

